# Were Women Staying on Track with Intermittent Preventive Treatment for Malaria in Antenatal Care Settings? A Cross-Sectional Study in Senegal

**DOI:** 10.3390/ijerph191912866

**Published:** 2022-10-08

**Authors:** Karen Zhang, Di Liang, Donglan Zhang, Jun Cao, Jiayan Huang

**Affiliations:** 1 Graduate School of Economics, University of Helsinki, Helsinki 00100, Finland; 2 School of Public Health, Global Health Institute, Fudan University, Shanghai 200032, China; 3 Division of Health Services Research, Department of Foundations of Medicine, Long Island School of Medicine, New York University, Mineola, NY 11501, USA; 4 Key Laboratory of National Health Commission on Parasitic Disease Control and Prevention, Key Laboratory of Jiangsu Province on Parasite and Vector Control Technology, Jiangsu Institute of Parasitic Diseases, Wuxi 214064, China

**Keywords:** intermittent preventive treatment in pregnancy, antenatal care, service readiness, cross-sectional study

## Abstract

A significant gap exists between high rates of antenatal care attendance and low uptake of intermittent preventive treatment in pregnancy with sulfadoxine-pyrimethamine (IPTp-SP) in Senegal. This study aims to investigate whether IPTp-SP is delivered per Senegal’s national guidelines and to identify factors affecting the delivery of IPTp-SP at antenatal care visits. A secondary analysis was conducted using the 2014 and 2016 Senegal’s Service Provision Assessment. The study sample consists of 1076 antenatal care across 369 health facilities. Multiple logit regression models were used to estimate the probability of receiving IPTp-SP during the antenatal care visit based on prior receipt of IPTp-SP and gestational age during the current pregnancy. At an antenatal care visit, the probability of receiving IPTp-SP is 84% (95% CI = [83%, 86%]) among women with no IPTp-SP history and 85% (95% CI = [79%, 92%]) among women with one prior dose. Women who visit a facility in the top quintile of the proportion of IPTp trained staff have a nearly 4-fold higher odds of receiving IPTp compared to those who visit a facility in the bottom quintile (95% CI = [1.54, 9.80]). The dose and timing of IPTp-SP provided in antenatal care settings in Senegal did not always conform with the national guideline. More training for providers and patient engagement is warranted to improve the uptake of IPTp-SP in antenatal care visits.

## 1. Introduction

Pregnant women are among the most vulnerable populations to malaria infection. In 2020, approximately 34 million pregnant women were at risk of exposure to the *Plasmodium falciparum* parasite.[1] For pregnant women, especially, malaria infection is life-threatening and accounts for over 10,000 maternal and 200,000 neonatal deaths per year.[1,2] Other adverse outcomes include increased risks of maternal anemia, prematurity, and low birth weight. In 2020, it was estimated that malaria infection during pregnancy accounted for 819,000 low birthweight newborns in sub-Saharan Africa [1].

Intermittent preventive treatment in pregnancy (IPTp) is a core intervention recommended by the World Health Organization (WHO) for the prevention of malaria in pregnancy [1]. IPTp with sulfadoxine-pyrimethamine (SP) is safe in pregnancy and effective in reducing the risk of clinical malaria during pregnancy, maternal anemia, placental parasitemia, and low birthweight [3,4,5,6,7,8]. The WHO currently recommends that women receive at least three doses of IPTp-SP during pregnancy [1]. IPTp-SP can be administered as early as possible in the second trimester and given at monthly intervals during scheduled antenatal care visits (ANC) up to the time of delivery.

While 33 moderate-to-high transmission Sub-Saharan Africa (SSA) countries have adopted IPTp into their national malaria control guidelines, the gap between the actual implementation of IPTp and target coverage levels remains large. According to the WHO Malaria World Report (2021), 74% of pregnant women report using ANC at least once; yet, only 57% of pregnant women receive at least one dose of IPTp, which is far below the recommended target level (90%) [1]. It has been estimated that if 90% of pregnant women received at least three doses of IPTp, an additional 206,000 low birth weights would have been averted in these 33 countries [1]. Understanding how IPTp is delivered at health facilities is crucial to closing the gap between ANC utilization and IPTp coverage.

Countries such as Senegal have made great strides in strengthening their health care infrastructure and reducing malaria transmission. Senegal has an annual malaria incidence rate of 50.5 cases per 1000, ranking it in the lowest 33rd percentile among malaria-endemic countries in the African region [2]. The national malaria control policy in Senegal focuses on achieving universal coverage of IPTp, insecticide-treated nets (ITNs), indoor residual spraying (IRS), malaria diagnostic testing, and prompt treatment of malaria with artemisinin-based combination therapies (ACTs) [9,10]. IPTp is one of the country’s earliest malaria interventions, adopted in 2005 [11]. According to the 2013 Senegal National Strategic Plan for Malaria Control, all pregnant women shall receive at least two doses of SP after the onset of active fetal movements, with the first dose given from the 16th week of amenorrhea or the perception of active movements of the fetus and the second at least one month after the first dose [12,13]. In 2016, about 50% of pregnant women in Senegal received two or more SP doses (IPTp-SP2+), above the 44% average among African countries [2,14]. However, compared to its 96% ANC utilization rate (2016), Senegal still experienced a substantial gap between ANC service and IPTp provision [15]. In the 2016–2020 National Strategic Plan, the recommended IPTp-SP treatment was updated to at least three doses per the most recent WHO recommendation. As Senegal sets a goal to increase the coverage of IPTp to at least three doses of SP (IPTp-SP3+) (In 2012, WHO changed the recommended SP doses from two to three, but Senegal did not complete the new IPTp training on the new recommendation until 2016 [14]. It also did not update the national malaria treatment and prevention guideline based on the new recommendation until 2016 [16].) to ≥80%, understanding how IPTp is provided at ANC visits and the roles that health care providers play are important for timely receipt of IPTp [15].

Previous studies have shown that the coverage of IPTp was affected by factors at the health system level, facility level, provider level, and individual level. Key health system issues affecting the provision and uptake of IPTp included the discordance between malaria control and maternal health policies, human resource shortages, and medication stock-outs [17,18]. At the facility level, private facilities were less likely to administer IPTp than facilities managed by public authorities [19]. Women’s individual factors, such as older age, higher education level, more ANC visits, receiving health education information and HIV counseling during ANC, previous pregnancy complications, and history of pregnancy, were reported to be positively associated with the uptake of IPTp, while never being married, long distance to ANC facility, young age, and limited ANC visits, were found to be negatively associated with the uptake of IPTp [20,21,22]. However, fewer studies have focused on factors influencing the uptake of IPTp among women attending ANC [19,20]. To our knowledge, no previous study has examined factors affecting the uptake of IPTp in ANC settings in Senegal [23,24]. This study aims to investigate the key factors influencing IPTp-SP administration in Senegal. Data from the Service Provision Assessment (SPA), a comprehensive survey implemented by the Demographic Health Survey (DHS Program) to determine the quality of health service delivery [25], is used to collect information from health care workers and women utilizing ANC and IPTp services.

## 2. Materials and Methods

### 2.1. Data Source and Study Sample

This is a secondary analysis of cross-sectional data obtained from Senegal’s 2014 and 2016 SPA surveys. The SPA, a component of the DHS program, is a comprehensive nationally representative health facility-based survey that generates health service delivery data from low-to-middle income countries. The overall goal of SPA is to evaluate whether the overall availability and readiness of different health services in a country are in accordance with United States Agency for International Development’s and WHO’s service readiness indicators [26]. For SPA, health facilities are representatively sampled at the national level by facility type, ownership, and geographic regions, and sampling weights are derived from accounting for the complex survey design [25]. Standardized questionnaires are administered to measure the providers’ training, inventory and infrastructure, and availability of treatment guidelines. Survey administrators also observed provider-patient consultations to determine whether consultations generally followed the accepted standard of care protocol, using a standardized checklist. Exit interviews are administered to a convenience sample of patients shortly after the consultation to determine their overall understanding of the provider’s instructions and perception of the visit. In Senegal, SPA surveys are conducted annually, except for provider-client consultation observations and exit interviews which are conducted biannually. Further details of SPA surveys are provided elsewhere [25].

**Figure A1:**
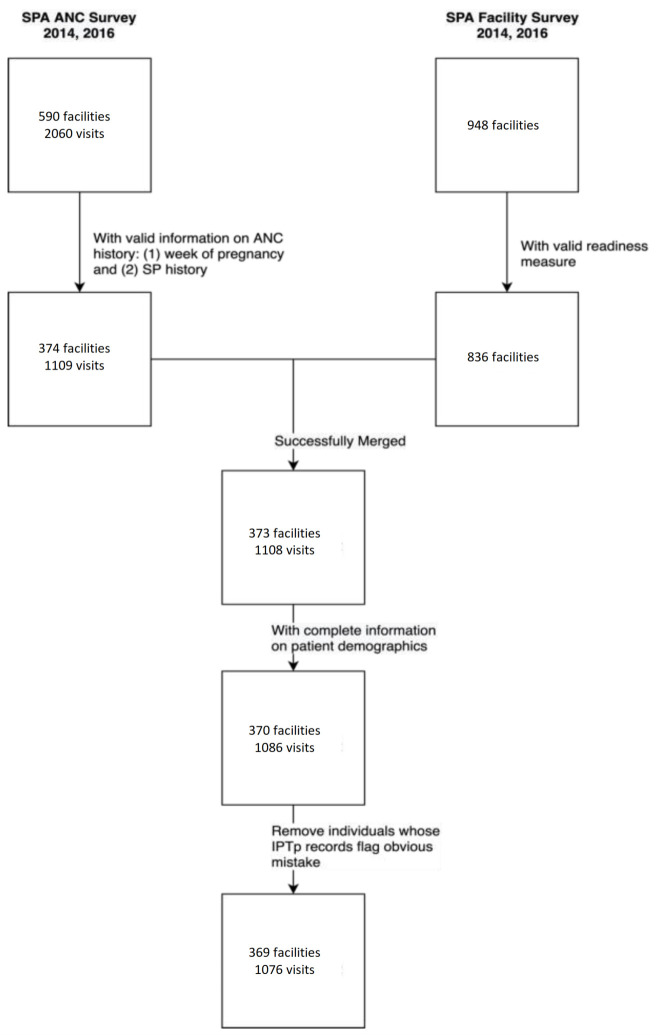
Flowchart of Sampled Health Facilities and Antenatal Care Visits from SPA Surveys Conducted in 2014 and 2016.

For our analyses, we combined data collected from the ANC consultation observation protocol, antenatal client exiting interview questionnaires, SPA facility questionnaires, and provider questionnaires administered during the 2014 and 2016 SPA survey waves. Only ANC visits with complete information for facility-level and individual-level characteristics are included in this complete-case analysis. As shown in Appendix A Figure A1, 984 visits are excluded due to missing data on pregnancy and/or IPTp history (951 visits), unsuccessful merging with health facility data (1 visit), incomplete information on pregnant women’s demographics (22 visits), and implausible IPTp records that occurred earlier than 8 weeks in pregnancy (10 visits).

### 2.2. Measures

This study examines the key factors associated with women receiving IPTp at their current ANC visit. The outcome variable is defined by whether a pregnant woman receives a dose of IPTp-SP during her current ANC visit in the ANC protocol observation survey of the SPA. Visits where providers “gave malaria prophylaxis medicine (SP) to client during the consultation” or “prescribed malaria prophylaxis medicine (SP) to client to obtain elsewhere” were coded as 1 and 0 otherwise.

The main explanatory variables of interest to predict the probability of receiving IPTp were gestational age and IPTp-SP history prior to the focal ANC visit (the ex-ante SP history). These are factors used by health care workers to determine whether a pregnant woman should receive IPTp-SP at her ANC visit [14,16,27]. Though gestational age is readily available in the client exit interview dataset, the prior number of IPTp doses needs to be constructed based on the ex-post IPTp-SP history. The client exit interview questionnaire documents the pregnant women’s gestational age and ex-post IPTp-SP history—number of IPTp-SP received after the completion of the focal ANC visit—on their ANC cards. Based on the ex-post IPTp-SP history, we reconstruct each pregnant woman’s ex-ante SP history depending on the number of SP prescriptions of her current visit and whether the provider was observed to update the medical card. Appendix A Table A1 illustrates the reconstruction algorithm. Facility-based characteristics were also explored to determine their association with receiving IPTp [28,29,30], including whether the national guidelines for the diagnosis, treatment, and prevention of malaria were physically posted at the health facility, whether the facility had at least one unit of valid SP, and the proportion of care providers trained on IPTp delivery that was working on the day of the survey [31]. Additional explanatory variables included maternal characteristics (e.g., age, education, parity, and whether the delivery was planned at the focal facility), region, year of survey, and a dummy variable indicating the rainy season (between June and October) in Senegal to account for the seasonal malaria transmission in Senegal.

### 2.3. Analysis

Using data from ANC visit protocol observations, we examine the probability that a pregnant woman would be prescribed SP during her ANC visit, conditioning on the number of SP dose(s) she has received prior to the focal visit. We first grouped ANC visits into three groups based on the ex-ante IPTp-SP history: (1) pregnant women who never had any SP dose before (IPTp-SP0), (2) pregnant women who had 1 SP dose before (IPTp-SP1), and (3) pregnant women who had at least 2 SP doses before (IPTp-SP2+). According to Senegal’s national malaria treatment and prevention guideline, IPTp-SP0 women ought to receive their first IPTp-SP dose at the ANC visit if they are at least 16 weeks of pregnant or active movements of the fetus are detected. Hence, in this category, we expect the probability of receiving IPTp-SP to be close to 100% for ANC visits after 16 weeks of pregnancy. Similarly, ANC visits of the second category should have a probability of receiving an IPTp-SP prescription near 100% conditioning on the last IPTp-SP dose at least a month earlier. Additional IPTp-SP doses are recommended by the WHO and Senegal’s national guidelines but are not required [2,16,27] (Current scientific evidence suggests that at least two doses of IPTp-SP are required to achieve optimal benefit in most women. However, some studies in HIV-infected pregnant women have demonstrated that monthly dosing of IPT, with most women receiving three to four doses, is necessary to achieve optimal benefit [6,7].). Therefore, the probabilities of receiving IPTp-SP treatment or prescription of ANC visits in the third category are not explicitly hypothesized but shed light on the interactions between pregnant women and care providers. For each group, we bin pregnant women by gestational age at every four weeks increment and report the share of receiving an SP prescription with a 95% confidence interval (CI).

We then use logit regression models to investigate factors that influence IPTp-SP delivery at ANC visits with two specifications. The first specification uses the categorical variable of IPTp-SP history as the main explanatory variable, adjusting for patient characteristics, facility characteristics, facilities’ malaria readiness measures, seasonality, and year and region fixed effects. The second specification adds the interaction between IPTp-SP history and week of pregnancy as additional explanatory variables. As logit regression models generate log odds (coefficients), we conducted post-estimation to generate the predicted probabilities of receiving IPTp-SP with other covariates at means as well as their 95% confidence intervals (CI) to help interpret the results. The sample is weighted by facility weights provided by the SPA assuming equal individual weights throughout to ensure that the contribution of facilities to the total is proportionate to their existence in the total. Sensitivity analyses were conducted with clustered standard errors at the regional level to account for the possibility of clustering at the regional level. All analyses are conducted using Stata17 (StataCorp, 2021; College Station, TX, USA: StataCorp LLC).

### 2.4. Ethical Considerations

No patients were involved in the process of generating the research question, designing the study, and implementing the study. No patients were asked to advise on writing up or interpretation of results. There are no plans to disseminate the findings to study participants or the relevant patient community.

## 3. Results

On average, pregnant women in our final sample were 27 years old and 27 gestational weeks (see Table 1). Among them, 42.8% had never received any SP dose before the ANC visit (see Appendix A Table A2), and 76% were pregnant for the first time. In addition, approximately 50% of pregnant women received some form of formal education. About 46% of visits took place in health facilities located in urban areas. Of the health facilities included in this study, 6% were hospitals, 13% were health centers, and 81% were health clinics. Eighty-eight percent of ANC visits in this study were conducted in government-owned facilities. About 92% of visits occurred in facilities equipped with malaria treatment and prevention guidelines. In comparison, only about 64% of visits occurred in facilities with unexpired SP in stock. Lastly, the proportion of staff trained on malaria IPTp guidelines that were on-site at the time of the survey averaged 31%. The regional distribution of ANC visits is presented in Appendix A Table A4.

**Figure 1:**
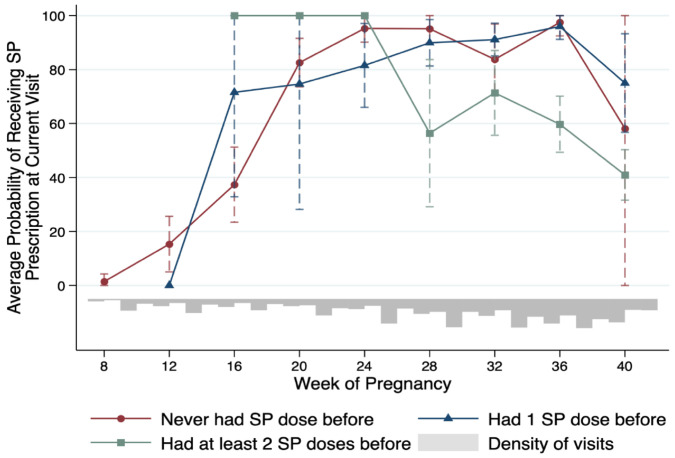
Mean Probability of Receiving IPTp Prescription at ANC Visits, by IPTp History. Note: The x-axis is the gestational age measured by month approximated by every 4-week interval. ANC visits in corresponding categories of IPTp history (color-coded) are collapsed at the same time interval to compute the mean probabilities (y-axis) and corresponding 95% confidence intervals. The bottom gray histogram plots the distribution density of the unconditional ANC visits along the week of pregnancy.

For the conditional probability of IPTp-SP prescription for each ANC visit category along with gestational age, we collapsed the ANC visits at the 4-week interval to compute the mean likelihood and 95% CI (see Figure 1). In addition to the color-coded probability curve of each IPTp-SP history category, we plot the distribution density of the unconditional ANC visits along the week of pregnancy at the time of the interview. Contrary to the national guideline, we find that pregnant women who previously never received IPTp-SP (red line) were not always prescribed SP. Specifically, only 37% (95% CI = [23%, 51%]) of pregnant women of 4-month pregnancy who needed the first IPTp-SP received the prescription. The probability increases to 83% (95% CI = [74%, 92%]) for women of 5-month pregnancy with the same IPTp-SP history but is still statistically significantly different from 100%. Although women further along in pregnancy had SP prescription probability not statistically different from 100%, the overall probability curve rejects the hypothesis that the first dose of IPTp-SP prescription is accordant to the national malaria treatment and prevention guideline.

The blue line visualizes the probability curve for ANC visits that required the second dose of IPTp-SP. Unlike the previous category, women of 4-month pregnancy or longer all had SP prescription probability that was not statistically different from 100%. However, this result is primarily due to the wide CIs of the sample means. The probability ranges from 71% in the fourth month of pregnancy to 96% in the ninth month.

Lastly, the green line depicts the probability curve for ANC visits that IPTp-SP prescription was optional according to the national guideline. Interestingly, ANC visits in this category had a high chance of receiving SP prescriptions. For instance, women who were 4–6 months pregnant in this category all received the optional SP prescriptions. Furthermore, among all women who already had the required 2 IPTp-SP doses, 60.8% received the SP prescriptions at the ANC visits.

We predict the probabilities that a pregnant woman receives an IPTp-SP prescription using our logit regression results (see Table 2; logit regression coefficients are reported in Appendix A Table A3, predicted probabilities). We use the IPTp-SP history of never having any SP dose before (IPTp-SP0) and its interaction term, when applicable, as the reference group. Without the interaction between IPTp-SP history and the week of pregnancy (Specification 1), the probability that IPTp-SP1 women receive SP prescriptions is not statistically different from IPTp-SP0 women. In comparison, IPTp-SP2+ women whose SP prescription is optional at the time of ANC visits are 0.08–0.48 times as likely to be prescribed with SP. The differences across IPTp-SP types are statistically significant. In Specification 2, we added the interaction terms between IPTp-SP types and weeks of pregnancy. An IPTp-SP0 patient has an 84% chance of receiving an SP prescription at mean gestational age and other covariates. The probability is similar for IPTp-SP1 women (85%) but reduces to 70% for IPTp-SP2 women, 61% for IPTp-SP3 women, and 42% for IPTp-SP4 women.

**Figure 2:**
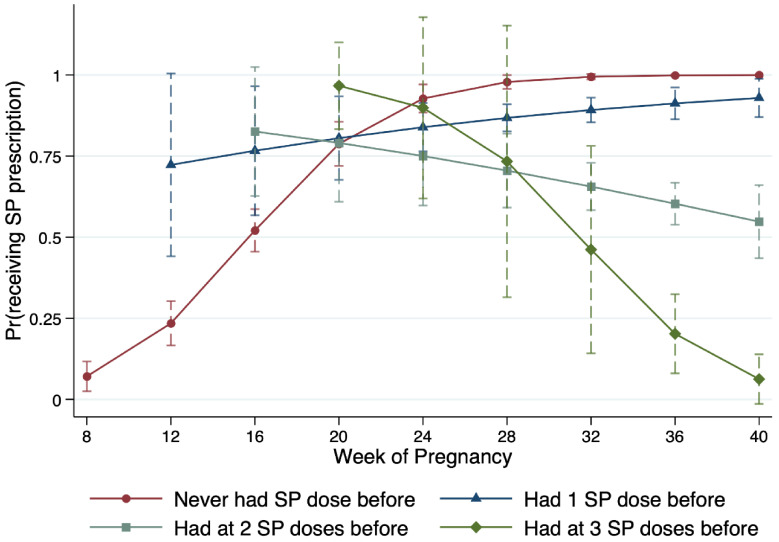
Predicted Probability of Receiving IPTp Prescription Varies by Gestational Age and IPTp History. Note: This figure presents the marginal mean probabilities and corresponding confidence intervals of IPTp prescription along the week of pregnancy. The x-axis is the gestational age measured at 4-week intervals. Color coding and line patterns are the same as in Figure 1 for the same category of IPTp history.

To further unpack the relationship between SP prescription, IPTp-SP history, and women’s gestational age, Figure 2 presents the marginal mean probability of SP prescription along the week of pregnancy. For illustration simplicity, we use the same color codes as in Figure 1 to distinguish between IPTp-SP types. For IPTp-SP0 (red circle) and IPTp-SP1 (blue triangle) women, the marginal mean probability of SP prescription increases the further along in pregnancy. The probability of a 16-week pregnant IPTp-SP0 patient who needs an SP prescription the most is only about 52%. This number is not only statistically significantly lower than the national recommendation but also statistically significantly lower than that of an IPTp-SP1 patient with a similar or longer pregnant length, as well as that of women whose IPTp-SP prescription is optional. For pregnant women whose IPTp-SP treatment is optional (IPTp-SP2+), the marginal mean probability of receiving an SP prescription decreases with gestational age.

Pregnant women’s characteristics also have a strong correlation with the probability of receiving IPTp-SP. Older women tend to have a lower probability of receiving IPTp-SP treatment or prescriptions. Women who are pregnant for the first time are more likely to receive SP but only significant at a 10% level. Among facility characteristics, we find facility location (rural vs. urban) does not significantly impact IPTp-SP provision. Different types of hospitals also have similar effects, except that providers in private for-profit facilities shrink the likelihood of treating with or prescribing IPTp-SP by a factor of 0.05 compared to providers in other types of facilities (Odds ratio is calculated using logit regression coefficients.). Lastly, a higher share of care providers who ever received IPTp training is positively correlated with the likelihood of IPTp-SP treatment and prescription.

The sensitivity analyses with clustered standard errors at the regional level showed similar results to the main findings (results not shown).

## 4. Discussion

Despite the national guideline requiring care providers to prescribe at least two doses of IPTp-SP (Because Senegal issued the minimum three doses of IPTp-SP guideline and completed the corresponding training in 2016, we refer to the earlier IPTp-SP2+ requirement in the discussion, which was in effect during the data collection of our analysis sample (SPA 2014, 2016).) to pregnant women at scheduled ANC visits, a significant proportion of pregnant women in Senegal did not receive IPTp-SP prescriptions per the guideline. Specifically, IPTp-SP0 pregnant women who are the prime target of IPTp intervention have a chance as low as 52% to receive their first IPTp-SP dose at ANC visits in the 4th month of pregnancy (84% throughout the course of pregnancy). A similar gap also presents among the IPTp-SP1 women, who only have an 85% chance on average to be prescribed the second required SP dose by ANC providers throughout the course of pregnancy.

Notably, this gap could not be explained by the lack of proper infrastructure for malaria services at the facility level. The national malaria treatment and prevention guidelines are available to 92% of facilities (Table 1). There is also evidence suggesting that the national guideline, is to some extent, binding for care providers. The probability of receiving the first two IPTp-SP doses at ANC visits is statistically higher than those of additional doses. The positive modification effect by gestational age among the IPTp-SP0 and IPTp-SP1 women also accredits the achievement of current interventions. Moreover, though only about 57.4% of facilities have unexpired SP in stock (Table 1), the availability of SP does not significantly predict a woman’s chance of receiving an IPTp-SP at her ANC visit. Previous literature often cited medication stock-outs as a crucial factor affecting the provision and uptake of IPTp [17,18]. Our findings suggested that the mere presence of medication was not enough to promote the uptake of IPTp.

The positive association between a pregnant woman’s accumulative IPTp-SP history and her likelihood of receiving an IPTp-SP at an ANC visit during the second trimester highlights the importance of the interaction between care providers and pregnant women in improving IPTp-SP delivery and broadly the prevention of malaria in pregnancy. On average, women who never received IPTp-SP before have a 75% chance of receiving an IPTp-SP prescription at their second trimester ANC visits. The probability increases to 80% for IPTp-SP1 women, 78% for IPTp-SP2 women, and 94% for IPTp-SP3 women (results not shown). Although IPTp-SP history is an important reference for providers to prescribe SP in ANC settings, how IPTp-SP history predicts the uptake of IPTp-SP was rarely documented in previous literature. Although the SPA surveys do not document the interactions leading to whether or not to provide IPTp-SP, the higher likelihood among pregnant women to whom the IPTp-SP is optional suggests IPTp history-dependent disparity. This disparity might possibly be explained by the characteristics of care providers and pregnant women. For instance, pregnant women who had received IPTp-SP before could be more aware of the risk and the adverse consequence of malaria infection; thus, they could be more likely to be proactive and initiate the conversation regarding IPTp-SP with ANC providers. Conversely, pregnant women with no or low IPTp-SP history may be less aware of the importance of IPTp-SP and rely on ANC providers to complete the required IPTp-SP doses. Further data collection and research would be warranted to examine those hypotheses.

After the IPTp intervention has been adopted in Senegal for nearly a decade, converting guideline recommendations at the policy level to care delivery in the field remains challenging. These findings raised the question: what steps can further close the gaps to the IPTp policy targets? As Crawley et al. pointed out, collaboration with reproduction health programs is the key step in implementing a malaria prevention policy targeting pregnant women at risk. [32] One area of actionable improvement is malaria IPTp training. For each sampled facility, only an average of 31% of care providers received malaria IPTp training in the past 24 months. At the facility level, moving from the bottom quintile in the share of IPTp trained staff to the top quintile is associated with a nearly four-time higher odds ratio (odds ratio is calculated using logit regression coefficients) (CI = [1.54, 9.80]) of providing IPTp-SP to pregnant women at the ANC visit. Emphasis on malaria IPTp training among ANC providers, particularly during the rainy season, would be an effective step to narrow the gap between IPTp policy-making and care delivery [33,34]. As Senegal moves from a minimum of two IPTp-SP doses to a three doses requirement, updating training programs and enforcing regular care provision assessments are necessary to ensure a smooth transition to the latest IPTp policy goal as well as future intervention upscaling [14].

While improving training for the provider side is necessary, dissemination of IPTp education among pregnant women such that more women at risk are aware of the IPTp-SP as an integrated part of ANC will help accelerate the improvement of IPTp-SP provision [35,36,37]. In addition, according to the updated WHO guidelines for malaria, other delivery methods (such as community health workers) could be explored to close the gap in ANC-based delivery of IPTp-SP [38]. These community-based services might be particularly helpful in reaching women who live far from ANC facilities.

This study has several limitations. First, our results draw from a cross-sectional analysis using a convenience sample. They are thus prone to non-response bias if pregnant women who participate in the survey differ from those who do not. Moreover, with insufficient panel data to track individual pregnant women’s ANC and IPTp-SP history, we can only draw an association, rather than temporal causality, between the likelihood of receiving IPTp-SP at ANC visit and explanatory variables. Particularly, the time interval between receiving the last IPTp-SP and this ANC visit is unknown in the SPA data. As a result, it is possible that not all women with at least one IPTp-SP dose before need to be prescribed SP at this ANC visit. However, women with one prior IPTp-SP dose had SP prescription probability not statistically different from 100% starting from their 4-month pregnancy. Thus, the omitted variable of time to the last IPTp-SP should not be a major threat to our findings. Other omitted variables due to data limitations (such as the distance to the ANC facility) could also potentially threaten the findings.

Second, we use IPTp-SP treatment or prescription by care providers to approximate IPTp-SP administration. This approximation may be problematic if the patient compliance level is low, which would negatively bias our estimations, and is likely to have a differential effect on IPTp history [39,40]. Thus, our results shall be interpreted as the best-case scenario probabilities. Further research is needed on IPTp compliance and its interaction with IPTp provision.

Third, we recover the IPTp-SP history for each interviewed pregnant woman based on the interviewers’ observations and records from pregnant women’s ANC cards, assuming that the ANC cards were updated timely and accurately. Although we remove observations with IPTp records that occurred too early in pregnancy (<8 weeks), recall bias/measurement error could bias our results away from the null. On the other hand, if those erroneous observations are a truthful reflection of IPTp history, they raise a concern about over-provision. Future IPTp policy shall address this issue to avoid adverse outcomes such as SP resistance.

## 5. Conclusions

This study shows that the dose and timing of IPTp-SP provided in antenatal care settings in Senegal did not always conform with the national guideline. Specifically, women with no previous IPTp-SP history have a chance significantly lower than 100% to receive the first IPTp-SP prescription at an ANC visit. More research on the interaction between care providers and patients is warranted to explain this phenomenon further. Moreover, more training for care providers could be integrated into future interventions aiming at improving IPTp-SP uptake.

## Figures and Tables

**Table 1:** Summary Statistics of Pregnant Women (weighted N = 997).

Variables	Mean/N	SD/%
Characteristics of Pregnant Women in Sample		
Age	26.7	6.5
Gestational age (week)	27.3	8.9
1st pregnancy	757	75.9
Ever attended school	496	49.8
Malaria Service Readiness		
Malaria guideline available	916	91.9
Valid SP in stock	572	57.4
% of staffs trained with malaria IPT	30.9	23.0
Other Characteristics of Facilities Visited		
Location		
Urban	458	45.9
Rural	540	54.1
Facility type		
Hospital	55	5.6
Health center	131	13.2
Clinic	811	81.3
Managing authority (ownership)		
Government/public	878	88.1
NGO/Private not-for-profit	54	5.4
Private for-profit	37	3.7
Mission/faith-based	28	2.8

Notes: Pregnant women sample pools over Senegal SPA facility survey data, antenatal care questionnaires 2014 and 2016. Three variables of malaria service readiness are calculated by the author using questions from the SPA facility survey and the SPA provider survey. N stands for number, and SD stands for standard deviation. Results were adjusted by survey weights.

**Table 2:** Predicted Probability of Receiving IPTp Prescription at Current ANC Visit, Main Results from Logit Regressions.

	Specification 1													
IPTp History	IPTp-SP0		IPTp-SP1			IPTp-SP2			IPTp-SP3			IPTp-SP4		
	Mean	95% CI	Mean	95% CI		Mean	95% CI		Mean	95% CI		Mean	95% CI	
Predicted Prob.	0.823	[0.799, 0.846]	0.784	[0.736, 0.831]		0.391	[0.328, 0.454]		0.065	[0.019, 0.110]		0.193	[−0.010, 0.397]	
	Specification 2													
IPTp History	IPTp-SP0		IPTp-SP1			IPTp-SP2			IPTp-SP3			IPTp-SP4		
	Mean	95% CI	Mean	95% CI		Mean	95% CI		Mean	95% CI		Mean	95% CI	
Predicted Prob.	0.844	[0.827, 0.861]	0.854	[0.792, 0.916]		0.699	[0.605, 0.793]		0.608	[0.419, 0.796]		0.415	[−0.001, 0.831]	
Wk Pregn	Mean	95% CI	Mean	95% CI		Mean	95% CI		Mean	95% CI		Mean	95% CI	
2nd mo.	0.071	[0.025, 0.117]	NA			NA			NA			NA		
3rd mo.	0.235	[0.166, 0.303]	0.723	[0.441, 1.005]		NA			NA			NA		
4th mo.	0.521	[0.455, 0.587]	0.767	[0.568, 0.965]		0.826	[0.627, 1.024]		NA			NA		
5th mo.	0.788	[0.720, 0.855]	0.805	[0.676, 0.934]		0.790	[0.609, 0.972]		0.967	[0.833, 1.100]		NA		
6th mo.	0.927	[0.884, 0.971]	0.839	[0.764, 0.914]		0.750	[0.597, 0.903]		0.899	[0.620, 1.178]		0.592	[−0.445, 1.629]	
7th mo.	0.978	[0.957, 1.000]	0.868	[0.826, 0.909]		0.705	[0.591, 0.819]		0.734	[0.315, 1.152]		0.382	[−0.273, 1.036]	
8th mo.	0.995	[0.987, 1.003]	0.892	[0.854, 0.930]		0.656	[0.583, 0.729]		0.462	[0.142, 0.782]		0.201	[−0.040, 0.443]	
9th mo.	0.999	[0.997, 1.001]	0.912	[0.863, 0.961]		0.603	[0.538, 0.668]		0.202	[0.080, 0.324]		0.088	[−0.054, 0.231]	
10th mo.	1.000	[0.999, 1.000]	0.929	[0.870, 0.988]		0.548	[0.435, 0.661]		0.063	[−0.014, 0.139]		0.034	[−0.084, 0.151]	

Notes: The predicted probability of receiving an IPTp prescription uses estimates based on prior IPTp history and gestational age in months from the logit regressions (reported in Table A3). Confidence intervals are calculated using standard errors adjusted for survey design. Specification 1 regresses the outcome variable on the categorical variable of women’s IPTp history and facility characteristics, women’s demographics, and other controls (see main text). Specification 2 adds the interactions between the IPTp history and women’s gestational age at the visit. CI stands for the confidence interval. NA stands for “not applicable.” The significance level is 0.05.

## Data Availability

Data are available in a public, open-access repository. Available at https://dhsprogram.com/ (accessed on 1 January 2021).

## List of Abbreviations

ACTs	artemisinin-based combination therapies
ANC	antenatal care
CI	confidence interval
DHS	Demographic Health Survey
IPTp	intermittent preventive treatment in pregnancy
IPTp-SP0/IPTp-SP1/IPTp-SP2/IPTp-SP3+	having had zero/one/two/at least three doses of SP
IRS	indoor residual spraying
ITNs	insecticide-treated nets
SD	standard deviation
SP	sulfadoxine-pyrimethamine
SPA	Service Provision Assessment
SSA	Sub-Saharan Africa
WHO	World Health Organization

## Appendix A

**Table A1:** IPTp History Recovery.

		Recovered # of IPTp Doses Before the Current Visit (ex-ante)			
		(1)		(2)	
ANC Card Record: Observed Total IPTp Doses at the Exit Interview (Ex-post)		Provider Did Not Prescribe SP or Update the Card (33%)		Provider Prescribed SP and Wrote on the Card (67%)	
IPTp Dose (e.p.)	%	IPTp Dose (e.a.)	%	IPTp Dose (e.a.)	%
0	16%	0	47%	N/A	N/A
1	31%	1	12%	0	41%
2	33%	2	28%	1	35%
3	18%	3	12%	2	21%
4	2%	4	2%	3	2%
Total N. visits		355		721	

Notes: The leftmost column tabulates the total ANC visits by the number of IPTp doses recorded in women’s ANC cards and observed in the exit interview after the ANC visits. Those observations are referred to as ex-post (e.p.) IPTp dose (see main text). Columns (1)–(2) show how the number of IPTp doses before the focal ANC visit (ex-ante, or e.a.) was recovered based on providers’ actions. Column (1) shows ANC visits where the providers did not proscribe SP or were not observed to update clients’ ANC cards. For those 355 observations, the ex-ante IPTp doses are the same as the ex-ante doses. Column (2) shows ANC visits where the providers proscribed SP and wrote on the clients’ ANC cards. For those observations, the ex-ante IPTp doses are ex-post IPTp minus 1. The bottom row reports the total number of ANC visits included in the sample and the split between the two categories. Numbers may not sum to 100% due to rounding.

**Table A2:** Summary Statistics of Pregnant Women’s IPTp History and Gestational Age.

	IPTp-SP0	IPTp-SP1	IPTp-SP2	IPTp-SP3	IPTp-SP4	(row N)
≤8 weeks	100.0%	0.0%	0.0%	0.0%	0.0%	35
9–12 weeks	99.2%	0.8%	0.0%	0.0%	0.0%	62
13–16 weeks	89.5%	10.6%	0.0%	0.0%	0.0%	56
17–20 weeks	97.0%	2.5%	0.0%	0.6%	0.0%	76
21–24 weeks	69.7%	26.4%	3.9%	0.0%	0.0%	117
25–28 weeks	49.0%	40.8%	8.0%	0.0%	2.1%	148
29–32 weeks	20.5%	50.2%	27.5%	0.3%	1.5%	157
33–36 weeks	9.2%	34.5%	46.6%	9.3%	0.4%	192
≤37 weeks	1.0%	17.3%	60.9%	19.1%	1.7%	154
Column N	427	271	243	48	9	997
(%)	42.8%	27.2%	24.3%	4.8%	0.9%	100%

Notes: Pregnant women sample pools over Senegal SPA facility survey data, antenatal care questionnaires 2014 and 2016. N stands for number. Results were adjusted by survey weights.

**Table A3:** Logit Regression Results, Coefficients.

	Logit, Coefficient					
	(1)			(2)		
	Coeff.	95% CI		Coeff.	95% CI	
Main Effects:						
[Reference] 0 dose	-	-	-	-	-	-
1 SP dose	−0.437	[−1.068	0.195]	6.612 ***	[3.318	9.907]
2 SP doses	−3.435 ***	[−4.247	−2.622]	9.456 ***	[5.895	13.017]
3 SP doses	−6.501 ***	[−7.804	−5.199]	18.166 **	[6.729	29.602]
4 SP doses	−4.913 ***	[−6.918	−2.908]	13.336	[−5.561	32.233]
[Reference] 0 dose X Week of pregnancy				-	-	-
1 SP dose X Week of pregnancy				−0.324 ***	[−0.457	−0.190]
2 SP doses X Week of pregnancy				−0.472 ***	[−0.603	−0.340]
3 SP doses X Week of pregnancy				−0.776 ***	[−1.101	−0.451]
4 SP doses X Week of pregnancy				−0.673 **	[−1.242	−0.104]
Patient Characteristics:						
Week of pregnancy	−0.029 **	[−0.058	−0.001]	−0.042 **	[−0.076	−0.008]
Age	0.198 ***	[0.157	0.238]	0.398 ***	[0.302	0.494]
First time pregnancy	0.378	[−0.138	0.894]	0.462	[−0.127	1.051]
Ever attended school	−0.223	[−0.614	0.167]	−0.177	[−0.591	0.238]
Will delivery here	0.202	[−0.249	0.654]	0.455	[−0.046	0.957]
Facility Characteristics:						
[Reference] Urban	-	-	-	-	-	-
Rural	0.624 **	[0.025	1.223]	0.374	[−0.260	1.009]
[Reference] Hospital	-	-	-	-	-	-
Health center	0.331	[−0.480	1.141]	0.589	[−0.352	1.531]
Clinic	0.158	[−0.680	0.995]	0.406	[−0.513	1.324]
[Reference] Public	-	-	-	-	-	-
NGO/private not-for-profit	−0.392	[−1.181	0.396]	−0.518	[−1.501	0.465]
Private for-profit	−2.244 ***	[−3.510	−0.977]	−2.996 ***	[−4.487	−1.504]
Mission/faith-based	0.141	[−1.525	1.806]	0.034	[−1.166	1.235]
Malaria Readiness:						
Malaria guideline available	0.125	[−0.563	0.814]	0.021	[−0.797	0.838]
SP in stock	0.360	[−0.105	0.825]	0.291	[−0.227	0.809]
Share of staff with IPTp training						
(Reference) 1st quintile	-	-	-	-	-	-
2nd quintile	0.001	[−0.731	0.722]	−0.093	[−0.783	0.598]
3rd quintile	−0.249	[−0.796	0.299]	−0.129	[−0.679	0.421]
4th quintile	0.470	[−0.173	1.112]	0.415	[−0.256	1.085]
5th quintile	1.122 **	[0.327	1.918]	1.357 ***	[0.431	2.282]
Others:						
Rainy season (Jan.-Jun.)	0.275	[−0.249	0.800]	0.483	[−0.058	1.023]
Constant	−3.729 ***	[−5.270	−2.188]	−7.188 ***	[−9.318	−5.058]
Year FE	Yes			Yes		
Region FE	Yes			Yes		
Observations	1076			1076		

Note: Specification 1 regresses the outcome variable on the categorical variable of women’s IPTp history and facility characteristics, women’s demographics, and other controls (see main text). Specification 2 adds the interactions between the IPTp history and women’s gestational age at the visit. Confidence intervals are calculated using standard errors adjusted for survey design. CI stands for the confidence interval. ** *p* < 0.01; ****p* < 0.001.

**Table A4:** Distribution of the Analysis Sample at the Regional Level.

	Analysis Sample	
	N	%
Dakar	242.00	24.3%
Diourbel	45.19	4.5%
Fatick	141.80	14.2%
Kaffrine	9.17	0.9%
Kaokack	33.27	3.3%
Kedougou	2.62	0.3%
Kolda	4.72	0.5%
Louga	67.53	6.8%
Matam	13.14	1.3%
Saint louis	39.84	4.0%
Sediou	77.32	7.8%
Tambacounda	74.05	7.4%
Thies	134.00	13.4%
Ziguinchor	112.40	11.3%
Total	997	100.0%

Note: Pregnant women sample pools over Senegal SPA facility survey data, antenatal care questionnaires 2014 and 2016. N stands for number. Results were adjusted by survey weights.
